# Metallothionein regulates intracellular zinc signaling during CD4^+^ T cell activation

**DOI:** 10.1186/s12865-016-0151-2

**Published:** 2016-06-02

**Authors:** James M. Rice, Adam Zweifach, Michael A. Lynes

**Affiliations:** Department of Molecular and Cell Biology, University of Connecticut, 91 North Eagleville Road, Unit 3125, Storrs, CT 06269 USA; Present address: Vascular Biology Program, Harvard Medical School, Boston Children’s Hospital, 300 Longwood Ave., Boston, 02115 MA USA

**Keywords:** Metallothionein, Zinc signal, CD4^+^ T helper cell, p38 MAPK, Tr1, Redox, T cell receptor

## Abstract

**Background:**

The ultra-low redox potential and zinc binding properties of the intracellular pool of mammalian metallothioneins (MT) suggest a role for MT in the transduction of redox signals into intracellular zinc signals. Increased expression of MT after exposure to heavy metals, oxidative stress, or inflammatory cytokines leads to an increased intracellular redox-mobilizable zinc pool that can affect downstream zinc-sensitive signaling pathways. CD4^+^ T helper cells are poised to be influenced by MT transduced zinc signaling because they produce intracellular reactive oxygen species following activation through the T cell receptor and are sensitive to small changes in intracellular [Zn^2+^].

**Results:**

MT expression and intracellular [Zn^2+^] are both increased during primary activation and expansion of naïve CD4^+^ T cells into the Tr1 phenotype in vitro. When Tr1 cells from wildtype mice are compared with congenic mice lacking functional *Mt1* and *Mt2* genes, the expression of intracellular MT is associated with a greater increase in intracellular [Zn^2+^] immediately following exposure to reactive oxygen species or upon restimulation through the T cell receptor. The release of Zn^2+^ from MT is associated with a greater increase in p38 MAPK activation following restimulation and decreased p38 MAPK activation in MT knockout Tr1 cells can be rescued by increasing intracellular [Zn^2+^]. Additionally, IL-10 secretion is increased in MT knockout Tr1 cells compared with wildtype controls and this increase is prevented when the intracellular [Zn^2+^] is increased experimentally.

**Conclusions:**

Differences in zinc signaling associated with MT expression appear to be a result of preferential oxidation of MT and concomitant release of Zn^2+^. Although zinc is released from many proteins following oxidation, release is greater when the cell contains an intracellular pool of MT. By expressing MT in response to certain environmental conditions, CD4^+^ T cells are able to more efficiently release intracellular zinc and regulate signaling pathways following stimulation. The link between MT expression and increased zinc signaling following activation represents an important immunomodulatory mechanism of MT and illuminates the complex role MT plays in shaping immune responses.

**Electronic supplementary material:**

The online version of this article (doi:10.1186/s12865-016-0151-2) contains supplementary material, which is available to authorized users.

## Background

Metallothioneins (MT) are low molecular weight, high cysteine content proteins that are expressed in most mammalian cells. Their upregulation in response to increases in intracellular zinc ion concentration ([Zn^2+^]_i_), reactive oxygen species (ROS), pro-inflammatory cytokines, and as part of proliferation and differentiation [1] suggests that MT may play an important role during the development of immune responses. Specifically, we hypothesize that upregulated MT expression can affect both intracellular redox and zinc signaling events that occur during CD4^+^ T helper cell activation. The release of zinc ions by MT in response to redox signaling may play a role in immune responses by directly influencing zinc signaling pathways during CD4^+^ T helper cell activation.

The intracellular labile zinc concentration has recently been recognized as an important component of T cell activation [2]. Modest increases in [Zn^2+^]_i_ (in the range of 200pM–1nM) can have profound effects on signaling pathways originating from the T cell receptor (TcR) [3], cytokine receptors [4, 5] and toll-like receptors [6]. Increases in [Zn^2+^]_i_ inhibit the activity of selected intracellular kinases [7–9] and phosphatases [10, 11] which, in turn, affect STAT and MAPK signaling networks and their associated transcription factor activity. Regulating [Zn^2+^]_i_, therefore, has the potential to dramatically influence the development of CD4^+^ T cell effector function [12–14] and diseases that arise from abnormal CD4^+^ T cell activity.

MT binds and releases zinc under physiologically relevant conditions and, in concert with the families of cell-specific zinc importers (Zip) and exporters (ZnT), is part of a network that tightly controls intracellular [Zn^2+^]_I_ [15]. The intracellular pool of MT is a mixture of oxidized, reduced and zinc bound forms of the protein [16, 17], and the dynamic equilibrium of these forms regulates zinc availability in response to intracellular ROS. Interestingly, zinc release from MT is possible even in the reducing environment of the cytosol through reactions with selenium redox catalysts [18, 19]. MT contains 20 cysteine residues which impart a very low redox potential (−366 mV), making it a preferential target for oxidation when compared with other free thiols including glutathione (GSH) [20, 21]. Of the seven zinc binding sites in MT, three have a comparatively weak metal binding affinity (logK = 10–7.7) [22] which facilitates zinc release upon thiol oxidation and results in a downstream effect on enzyme activity [23]. This property uniquely positions MT to transduce ROS signals into zinc signals within the narrow ranges of redox and [Zn^2+^]_i_ fluctuations that occur during CD4+ T cell activation.

Naïve CD4^+^ T cells express low levels of MT [24, 25]. Upon activation, [Zn^2+^]_i_ increases over time as a result of the release of zinc stored in vesicles into the cytoplasm [12] and from increased zinc import from the extracellular environment [3, 26]. This sustained zinc signal [27] induces the expression of MT which supports T cell proliferation [26]. During primary CD4^+^ T cell activation and differentiation, extracellular cytokines, including TNF-alpha, IL-1, IL-6, and IL-27 [28–30] and glucocorticoids [31], also influence the expression of MT and simultaneously drive T cell differentiation. Cells with an increased concentration of cytoplasmic MT are then positioned to more efficiently convert ROS signals into zinc signals [32] during subsequent activation events, which has the potential to affect CD4^+^ T helper cell function.

In this report, we confirm that MT expression following the primary activation of naïve CD4^+^ T cells is influenced by the cytokine IL-27 [30]. Furthermore, we show that expression of MT provides CD4^+^ T cells with a redox-sensitive pool of intracellular zinc that can be mobilized under conditions of oxidative stress or in response to intracellular ROS generation following signaling through the TcR. In the absence of functional *Mt1* and *Mt2* genes (MT ^*-/-*^)*,* the increase in [Zn^2+^]_i_ following redox signaling is reduced, and this results in decreased p38 activation in MT ^*-/-*^ cells which can be rescued by pharmacologically increasing [Zn^2+^]_i_. These results demonstrate that MT plays a role in CD4^+^ T cell activation by transducing ROS signals into an increased [Zn^2+^]_i_ that subsequently affects downstream effector function.

## Results

Activation and proliferation of CD4^+^ T cells is associated with an increase in the concentration of intracellular labile zinc ions ([Zn^2+^]_i_) [33] and the expression of metallothioneins (MT) [25]. Manipulating [Zn^2+]^_i_ [13, 34] or MT expression [30, 35] during activation affects cell signaling networks and cytokine secretion patterns. In elderly populations, a decreased ability to regulate increases in [Zn^2+^]_i_ following CD4^+^ T cell activation results in increased MT expression and altered T cell function [26, 36]. This suggests that zinc and MT are coordinately regulated during activation and this allows CD4^+^ T cells to respond appropriately in different environments.

**Fig. 1:**
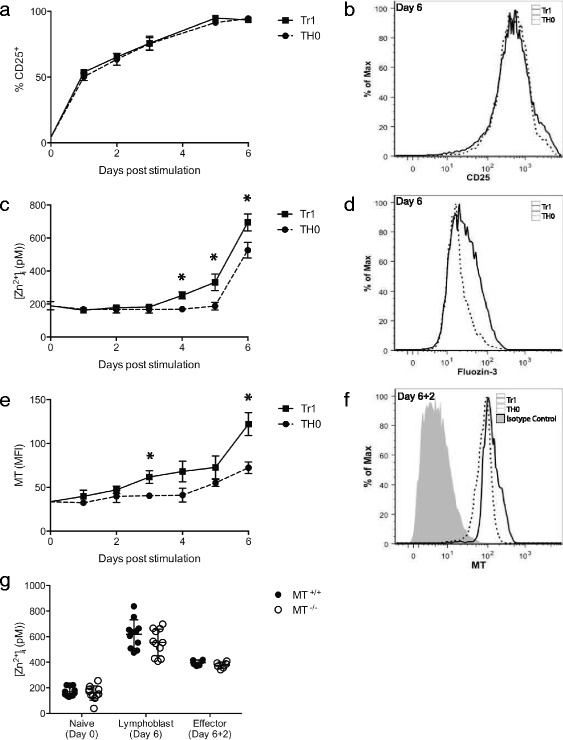
[Zn^2+^]_i_ and MT expression are regulated during CD4^+^ T helper cell differentiation and affected by IL-27. Mononuclear cells were isolated from spleens of C57BL/6 mice (*n* = 7) and stimulated for 6 days with anti-CD3 and anti-CD28 antibodies in the presence of IL-27 or without cytokines to induce a Tr1 or Th0 phenotype of CD4^+^ T cells, respectively. **a**, **b** CD25 expression, (**c**, **d**) intracellular [Zn^2+^] and (**e**, **f**) MT expression in the CD4^+^ T cell population were measured at 24-h intervals for 6 days. Histograms are representative of one Tr1 sample (*solid line*) and one Th0 sample (*dotted line*). **g** Mononuclear splenocytes from metallothionein knockout mice (MT^-/-^) (*n* = 11) or congenic wildtype controls (MT^+/+^) (*n* = 11) were cultured under Tr1 inducing conditions for 6 days and then rested for an additional 2 days. Intracellular [Zn^2+^] was measured on Day 0, Day 6, and Day 8 which corresponded to naïve, lymphoblast and effector stages of CD4^+^ T cell development, respectively. Error bars indicate standard deviation. *p < .05

To determine the degree to which CD4^+^ T cells regulate [Zn^2+^]_i_ and MT expression during activation and effector cell development, naïve CD4^+^ T cells were stimulated using anti-CD3 and anti-CD28 antibodies in the presence or absence of IL-27 to promote the development of Tr1 or Th0 phenotypes, respectively [37, 38]. In both culture conditions, expression of CD25 served as an activation marker and was increased by 24 h post-stimulation (Fig. 1a). After 6 days of culture, CD25 was expressed by >95 % of CD4^+^ T cells in both conditions, (Fig. 1b) indicating cell activation was not reduced in the absence of IL-27 signaling.

An increase in [Zn^2+^]_i_ in CD4^+^ T cells activated under Th0 inducing conditions was observed after 6 days (Fig. 1c). This increase followed the expression of CD25, indicating naïve CD4^+^ T cells maintain zinc homeostasis in the time period following initial activation events. In the presence of IL-27, [Zn^2+^]_i_ was increased by 4 days post activation (Fig. 1c) and after 6 days was significantly higher than in cells induced under Th0 conditions (Fig. 1d), suggesting a link between IL-27 signaling and the regulation of [Zn^2+^]_i_ during primary activation. Next, we measured intracellular MT expression during the same activation window. A modest increase in MT expression was observed 6 days after activation under Th0 inducing conditions while MT expression was increased significantly when cells were activated in the presence of IL-27 (Fig. 1e). This increased MT expression persisted for at least two days after cells were transferred and cultured in media alone (Fig. 1f), identifying a 48 h temporal window where altered MT expression could potentially play a role during CD4^+^ T cells re-activation. Interestingly, the increase in intracellular MT in naïve CD4^+^ T cells 3 days after activation preceded a measurable increase in [Zn^2+^]_i_, indicating MT induction did not require an increase in [Zn^2+^]_i_ under these culture conditions. However, the highest level of MT expression and [Zn^2+^]_i_ both occurred on day 6 (Fig. 1c, e), supporting the well-established link between increased [Zn^2+^]_i_ and MT induction.

To determine if MT gene dose affects intracellular zinc homeostasis during the development of Tr1 cells, the [Zn^2+^]_i_ in *Mt1Mt2* knockout cells (MT^-/-^) was compared with wildtype congenic cells (MT^+/+^) during naïve CD4^+^ T cell activation. The same pattern of [Zn^2+^]_i_ increase following activation was observed in both MT^+/+^ and MT^-/-^ CD4^+^ T cells at each of the stages of Tr1 cell differentiation (Fig. 1g), indicating that MT did not significantly affect intracellular labile zinc homeostasis under these activation conditions. [Zn^2+^]_i_ was reduced when proliferating lymphoblasts were resuspended in fresh media with no additional stimulation for 2 days, demonstrating that the increase in the [Zn^2+^]_i_ above 500 pM is transient and associated with activation and the lymphoblast phenotype.

**Fig. 2:**
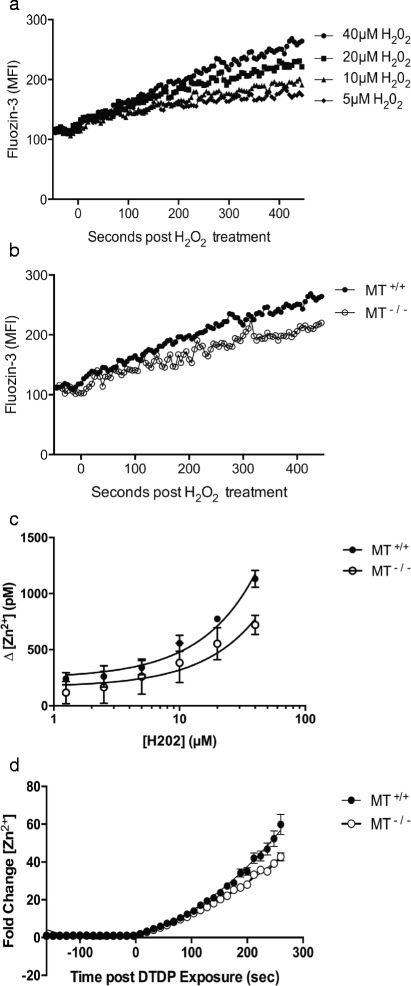
Metallothionein expression in Tr1 cells is associated with increased Zn^2+^ release following exposure to H_2_O_2_ or DTDP. Intracellular Zn^2+^ release was measured in Tr1 cells from metallothionein knockout mice (MT^-/-^) (*n* = 3) or congenic wildtype control mice (MT^+/+^) (*n* = 3) exposed to different concentrations of H_2_O_2_ to measure oxidant-induced Zn^2+^ release. **a** The release of intracellular Zn^2+^ was measured by an increase in fluozin-3 fluorescence in wildtype Tr1 cells before and after exposure to H_2_O_2_ [5–40 μM] or (**b**) for metallothionein knockout (MT^-/-^) Tr1 cells compared with wildtype controls exposed to H_2_O_2_ [40 μM] in one representative sample of each treatment is shown. **c** The increase in intracellular [Zn^2+^] after 7 min of H_2_O_2_ [1.25–40 μM] exposure was compared using linear regression. **d** The fold-increase in intracellular [Zn^2+^] after 100 μM DTDP exposure was compared using linear regression. Error bars indicate standard deviation

The increased intracellular pool of zinc-MT that is present after the development of the CD4^+^ Tr1 cell effector phenotype is a potential reservoir of zinc that can be mobilized during reactivation. We assessed whether this MT-bound zinc pool could be released in response to reactive oxygen species (ROS) and affect [Zn^2+^]_i_. Exposing CD4^+^ Tr1 cells to hydrogen peroxide (H_2_O_2_), a biologically relevant oxidant associated with T cell activation [39], for 7 min resulted in an increase in [Zn^2+^]_i_ in a dose dependent manner (Fig. 2a). This size and timing of this increase was unaffected by the addition of 20 μM ZnSO_4_ to the media during the time tested (Additional file 1: Figure S1), confirming that the increase in [Zn^2+^]_i_ was not due to an influx of extracellular zinc. Oxidant-induced release of zinc was significantly greater in MT^+/+^ CD4^+^ T cells than in MT^-/-^ CD4^+^ T cells during the initial 7 min of exposure when compared by linear regression analysis (*p* < .05) (Fig. 2b and c).

To confirm that the oxidant-releasable zinc was sequestered via a thiolate bond, the thiol-specific oxidant DTDP was added to cells and the release of zinc was measured over time. As with H_2_O_2_, the release of Zn^2+^ following DTDP exposure was greater in MT^+/+^ Tr1 cells than in MT^-/-^ Tr1 cells (*p* < .05) (Fig. 2d). The robust release of Zn^2+^ following either oxidant exposure, irrespective of MT gene dose, confirms that MT is not the only redox-sensitive mobilizable pool of intracellular zinc (Fig. 2d). When a pool of zinc-MT is present, however, exposure to ROS results in a faster and larger increase in intracellular [Zn^2+^]_I_, indicating that MT contributes to a more efficient transduction of an oxidant signal into a zinc signal compared with other zinc-bound proteins. This additional labile zinc has the potential to inhibit the activity of several important T cell protein tyrosine phosphatases [4, 10] and affect downstream cell signaling.

**Fig. 3:**
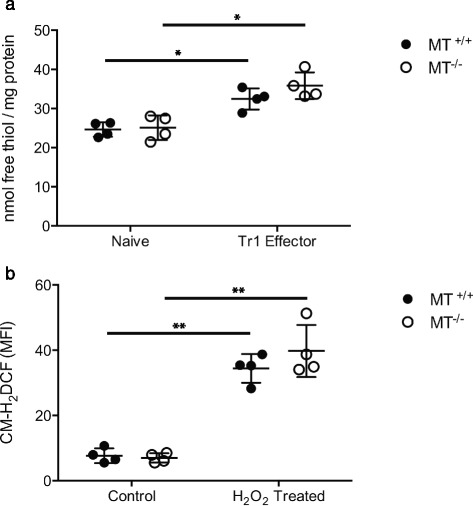
Metallothionein expression does not affect the total redox buffering capacity of Tr1 cells. CD4^+^ T cells from metallothionein knockout (MT^-/-^) (*n* = 4) or congenic wildtype control mice (MT^+/+^) (*n* = 4) were cultured for 0 (naïve) or 6 days in the presence of Il-27 (Tr1 effector). **a** Cell lysate was analyzed for the presence of free thiols by CPM assay and total protein concentration by BCA assay. **b** Tr1 cells were loaded with the oxidant sensitive probe CM-H_2_DCFDA. Fluorescence from the oxidized form of the probe was measured before and after H_2_O_2_ [50 μM] or PBS control exposure for 30 min to determine the intracellular redox buffering capacity. Bars indicate standard deviation. (*p < .05, **p < .01)

Global changes in intracellular redox buffering capacity also have the potential to affect oxidant-induced zinc release from protein thiols [40]. To determine if MT gene dose influences the total intracellular redox buffering capacity, total reduced cellular thiols were quantified in MT^-/-^ and wildtype control MT^+/+^ CD4^+^ naïve and effector T cells by CPM assay [41]. The concentration of reduced thiols was higher in CD4^+^ Tr1 effector cells compared with naïve lymphocytes (*p* < .05), but was not different between MT^-/-^ and MT^+/+^ Tr1 cells (Fig. 3a), indicating that the expression of MT did not significantly alter the total intracellular free thiol concentration. This is not unexpected, given that reduced glutathione exists in >1000 fold molar excess compared to MT in CD4^+^ T cells [25, 42]. Additionally, MT expression did not contribute to a significant difference in the total redox buffering capacity, as shown by the similar level of oxidation of the fluorescent probe CM-H_2_DCF in MT^-/-^ and MT^+/+^ Tr1 cells after exposure to H_2_O_2_ (Fig. 3b). These results suggest that MT is not a significant source of total intracellular free thiols under these cell culture conditions and that CD4^+^ T cells maintain their intracellular redox potential in the absence of MT. However, when MT is present as part of the intracellular pool of free thiols, cells maintain a greater zinc release potential in response to ROS. In this way, MT serves to amplify zinc signals that are transduced by an initiating oxidant signal without affecting total intracellular redox buffering capacity or the oxidant signal itself.

**Fig. 4:**
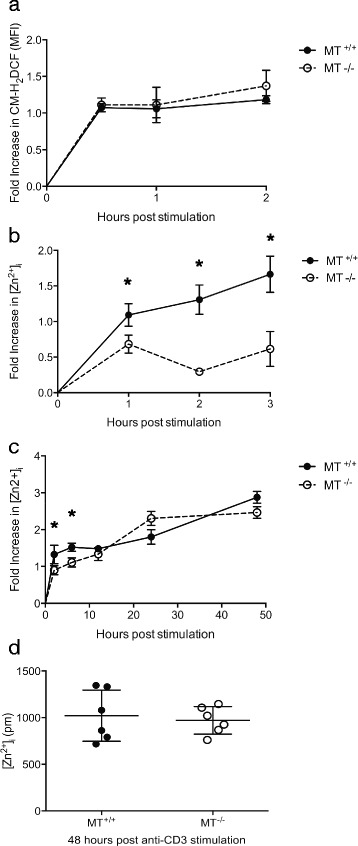
Metallothionein gene dose affects intracellular [Zn^2+^] in Tr1 cells following stimulation through the T cell receptor. Tr1 cells from metallothionein knockout (MT^-/-^, *n* = 4) or congentic wildtype control mice (MT^+/+^, *n* = 4) were loaded with CM-H_2_DCFDA and stimulated with anti-CD3 [10 μg/mL] cross-linked with anti-hamster IgG [10 μg/mL] for 0–120 min. **a** The fold change in CM-H_2_DCF fluorescence or (**b**) intracellular [Zn^2+^] in stimulated Tr1 cells compared with unstimulated control cells for each strain at 30, 60 and 120 min is shown. **c**, **d** MT^+/+^ (*n* = 6) and MT^-/-^ (*n* = 6) Tr1 cells were stimulated with plate-bound anti-CD3 [5 μg/mL] for 48 h. **c** The fold increase in intracellular [Zn^2+^] was measured at 4, 8, 12, 24 and 48 h. **d** The intracellular [Zn^2+^] of each sample after 48 h is shown. Error bars indicate standard deviation. (*p < .05)

Intracellular ROS generation is an important component of effector CD4^+^ T cell signaling following TcR activation [39, 43, 44] and is a requirement for effector function and expression of important regulatory molecules including Fas ligand (FasL). Hydroxyl radicals, superoxides, hydrogen peroxides, and nitric oxides [45], have all been shown to release Zn^2+^ from MT either directly [20, 21, 46], or indirectly via a mechanism where reduced glutathione (GSH) is oxidized to glutathione disulfide (GSSG), which then oxidizes MT [47]. To determine if ROS production is affected by the presence of MT, CD4^+^ Tr1 cells were stimulated with anti-CD3 cross-linked with anti-IgG, which is sufficient to stimulate ROS production in CD4^+^ T cells [43]. CD4^+^ Tr1 cells produced a robust intracellular ROS signal within 30 min of TcR stimulation (Fig. 4a) and ROS persisted for at least 2 h indicated by the oxidation of the CM-H_2_DCF probe. Oxidation of the probe after activation was not significantly higher in MT^-/-^ cells compared with MT^+/+^ control cells, further supporting the conclusion that MT is part of a larger intracellular redox-buffering network and is not essential for maintaining cellular redox buffering capacity.

Despite similar levels of ROS generation following activation, the increase in [Zn^2+^]_i_ after 1 h of stimulation was greater in MT^+/+^ Tr1 cells (Fig. 4b) compared with MT^-/-^ Tr1 cells. Similar to exogenous hydrogen peroxide exposure (Fig. 2), this increase was not affected by the addition of 20 μM ZnSO_4_ during the initial 3 h time period, indicating the difference in zinc levels was not due to a zinc influx from the extracellular environment (data not shown). The increase in [Zn^2+^]_i_ following reactivation of Tr1 cells with anti-CD3 continued for at least 48 h (Fig. 4c) and the peak [Zn^2+^]_i_ was higher during restimulation than during primary activation (Figs. 1d and 4d). After 8 h of restimulation, the increased [Zn^2+^]_I_ was no longer greater in MT^+/+^ Tr1 cells compared with MT ^-/-^ Tr1 cells. This suggests that while immediate increases in [Zn^2+^]_i_ following reactivation are affected by the release of Zn^2+^ from the intracellular stores, [Zn^2+^]_i_ 12 h after restimulation Zn^2+^ is predominantly regulated by ion transporters associated with T cell activation [12, 33]. Interestingly, CD4^+^ effector cells differentiated under Th1 conditions achieved an even higher [Zn^2+^]_i_ after 48 h restimulation compared with either Tr1 or Th0 cells, although there was no difference associated with MT genotype (Additional file 2: Figure S2). This suggests that different CD4^+^ T helper subsets regulate [Zn^2+^]_i_ differently to mediate effector function.

**Fig. 5:**
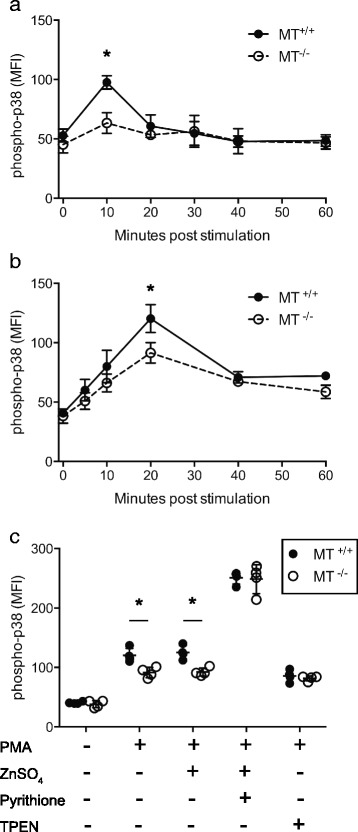
Metallothionein gene dose affects p38 MAPK activation in a Zn^2+^-dependent manner. Tr1 cells from metallothionein knockout (MT^-/-^) (*n* = 4) or wildtype control mice (MT^+/+^) (*n* = 4) were stimulated with (**a**) plate-bound anti-CD3 [5 μg/mL] or (**b**) 100 ng/mL PMA for 0–60 min. Cells were fixed, permeabilized and probed for the presence of phospho-p38. **c** During PMA induced activation, cells were treated with ZnSO_4_ [20 μM] with or without the zinc ionophore pyrithione, or with the zinc chelator (TPEN). Cells were fixed and permeabilized after 20 min and probed for and presence of phospho-p38. Error bars indicate standard deviation. (*p < .05)

Intracellular labile zinc can influence T cell signaling by directly affecting both the STAT [9, 48] and MAPK pathways [2, 4]. The co-stimulatory effect of Zn^2+^ on p38 MAPK signaling in CD4^+^ T cells has recently been observed following signaling through both the T cell receptor [7] and the IL-1 receptor [49]. During activation, relatively small increases in [Zn^2+^]_i_ can inhibit protein tyrosine phosphatase activity [3, 10], which results in increased phosphorylation of p38 MAPK [50]. Other MAPK proteins are also affected by [Zn^2+^]_i_ following stimulation through the TcR including ERK1/2, but we chose to investigate p38 because the inhibitory phosphatase that regulates p38 phosphorylation is sensitive to the picomolar increases in [Zn^2+^]_i_ that were observed when MT was present following restimulation of Tr1 cells. To determine if a co-stimulatory effect of Zn^2+^ on p38 activation is associated with a ROS-mediated release of Zn^2+^ from MT, phosphorylated p38 was measured in MT^+/+^ or MT^-/-^ Tr1 cells during re-stimulation through the TcR in vitro. Phospho-p38 levels peaked at 10 min post re-stimulation with anti-CD3 antibody, irrespective of MT gene dose (Fig. 5a). This increase was significantly higher in MT^+/+^ Tr1 cells compared with MT^-/-^ Tr1 cells and was not a result of differences in total p38 levels between the two strains (Additional file 3: Figure S3).

An association between MT gene dose and increased p38 activation was also observed when cells were stimulated with the protein kinase C activator PMA. Peak levels of phospho-p38 were observed after 20 min of PMA stimulation in both MT^+/+^ and MT^-/-^ Tr1 CD4^+^ cells and were again significantly higher in cells that express MT (Fig. 5b). PMA treatment did not appear to induce a zinc influx from the extracellular environment because p38 activation was not affected by increasing the extracellular [Zn^2+^] during stimulation with PMA (Fig. 5c). However, PMA does promote ROS generation [44] and a subsequent increase in [Zn^2+^]_i_ [51] in CD4^+^ T effector cells. This suggests zinc released from MT by ROS increases p38 activation and represents a novel mechanism for regulating p38 activation through expression of MT.

Differences in p38 activation mediated by MT were overcome with the addition of ZnSO_4_ together with the zinc ionophore pyrithione, indicating MT^-/-^ Tr1 cells were capable of achieving levels of p38 activation similar to MT^+/+^ cells when sufficient intracellular free zinc was present. Furthermore, differences in MT expression did not affect the activation of p38 when the available pool of intracellular labile zinc was removed by adding the zinc chelator TPEN (Fig. 5c). The observation that p38 activation was not affected by MT gene dose under conditions of similar intracellular zinc availability, via addition of a zinc specific ionophore or chelator, suggests that MT expression affects p38 activation through the regulation of available intracellular labile zinc.

**Fig. 6:**
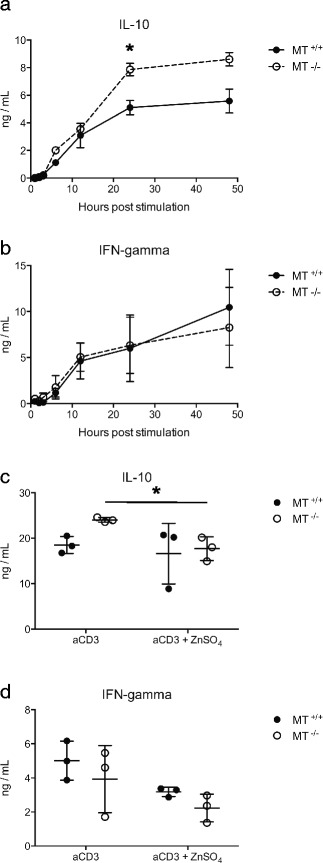
Release of the MT-dependent Zn2+ pool decreases IL-10 secretion but does not affect IFN-gamma release. Tr1 induced splenocyte populations from metallothionein knockout (MT^-/-^) (*n* = 3) or wildtype control mice (MT^+/+^) (*n* = 3) were restimulated with anti-CD3 [5 μg/mL] for 48 h. Supernatants were collected at different timepoints and analyzed for the presence of (**a**) IL-10 and (**b**) IFN-gamma by ELISA. **c** Tr1 cells from metallothionein knockout (MT^-/-^) (*n* = 3) or wildtype control mice (MT^+/+^) (*n* = 3) were pre-incubated with ZnSO_4_ [20 μM] (to increase intracellular [Zn^2+^]) or in media alone for 24 h before being restimulated with anti-CD3 [5 μg/mL] for an additional 24 h. Error bars indicate standard deviation. (*p < .05)

The downstream effects of increased [Zn^2+^]_i_ can include altered effector phenotype and cytokine secretion [9, 13, 48]. There are reports that MT gene dose can influence IL-10 expression, but these effects have not been consistently observed [30, 35]. To determine if MT gene dose affects the expression of cytokines, we measured secretion of IL-10 and IFN-gamma from MT^+/+^ and MT^-/-^ Tr1 cells. Secretion of IL-10 was detected in the media by 6 h post stimulation and was greater in MT^-/-^ Tr1 cell cultures compared with MT^+/+^ controls at 24 and 48 h post stimulation (Fig. 6a). Secretion of IFN-gamma during the same time period was not significantly affected by MT gene dose (Fig. 6b), suggesting that the influence of MT on IL-10 secretion is cytokine specific and not due to a more general effect on the secretion of all cytokines.

To determine if the elevated IL-10 secretion levels observed in MT^-/-^ cells were sensitive to [Zn^2+^]_i_, the resting zinc level was increased in Tr1 CD4^+^ cells by pre-incubating cells in media containing ZnSO_4_ [20 μM] for 24 h before restimulation (Additional file 4: Figure S4). This enabled us to test the effect of an increased [Zn^2+^]_i_ in Tr1 cells during the early activation window (0-6 h) where MT associated increases in the labile zinc pool had been observed (Fig. 4b and c). This concentration of ZnSO_4_ did not affect cell viability and in the presence of 10 % FBS (which buffers labile Zn^2+^ ions), is estimated to increase the extracellular labile [Zn^2+^] in media to 10 μM [34]. Increasing the [Zn^2+^]_i_ during restimulation significantly decreased IL-10 secretion from MT^-/-^ but not MT^+/+^ Tr1 cells (Fig. 6c). During the same time period, increasing [Zn^2+^]_i_ did not significantly affect IFN-gamma secretion, further supporting the conclusion that regulation of effector function by Zn^2+^ released from MT is cytokine specific. This is in line with previous studies which found that pre-incubation in 25 μM zinc aspartate reduced IL-2, IL-10, and IL-17 secretion following anti-CD3 stimulation in naïve splenocytes [13]. Our observations support this finding and suggest that MT mediated differences in IL-10 secretion are mediated through the regulation of [Zn^2+^]_I_ in CD4^+^ Tr1 cells.

## Discussion

The relationship between MT expression and immune function is complex. Both extracellular and intracellular pools of MT play important roles in modulating immune responses and influence a range of cellular behaviors including chemotaxis [52, 53], apoptosis [54], proliferation [55], differentiation [30, 56], and resistance to cellular stress [57] and infection [58]. Furthermore, MT expression is increased in many pathological conditions [59] including several types of cancer [60] and inflammatory auto-immune diseases [61] and significantly effects the development of both humoral [62] and cellular adaptive immune responses [35].

In this study, we have specifically investigated CD4^+^ Tr1 cell responses in vitro to assess the effect of intracellular MT expression on zinc signaling. The results of this study demonstrate that intracellular MT plays an important role as a signal transducer by converting ROS signals into a transient increase in labile [Zn^2+^]_i_ following Tr1 cell re-activation. The amplitude of this zinc signal is sufficient to regulate zinc-sensitive signaling pathways and influence effector function. The ability of MT to release free zinc ions in response to different types of cellular stress is well established. However, this is the first time, to our knowledge, that MT expression has been shown to strengthen the zinc signal resulting from T cell activation and have an effect on p38 MAPK signaling.

The relationship between p38 MAPK signaling and expression of IL-10 is poorly understood. While it has been reported that pharmacological inhibition of p38 activation down-regulates IL-10 expression [63, 64], much less is known about the effect of changes in p38 activation that are confined to the 30 min following stimulation, which were observed in CD4^+^ T cells that differed in MT expression. Our results indicate that reduced p38 activation in cells that do not express MT does not reduce IL-10 or IFN-gamma expression. It is therefore likely that other zinc sensitive signaling pathways including ERK1/2 [7], which have been shown to regulate IL-10 expression [65], may play a role during Tr1 cell restimulation and secretion of IL-10.

The control of intracellular labile zinc is a complex process where the active transport, sequestration, and release of zinc ions from intracellular thiols in response to different environmental stimuli are continuously regulated. The maintenance of zinc homeostasis and the development of normal T cell responses in CD4^+^ T cells from MT knockout mice suggest that MT is only part of a larger network of zinc regulation. However, recent evidence has revealed that even small changes in [Zn^2+^]_i_ can significantly affect signaling networks, especially those involving intracellular protein tyrosine phosphatases. This suggests that the contribution of MT in amplifying oxidant-induced zinc signals has downstream consequences, even in the presence of other regulators of zinc homeostasis.

The appropriate upregulation of MT expression in the presence of cytokines such as IL-27 and IL-6 provides a mechanism by which MT primes CD4^+^ T helper cells to differentially respond to ROS signals following re-activation and/or exposure to oxidative stress by transiently increasing the [Zn^2+^]_i_. The timing and magnitude of this amplified zinc signal are ideally positioned to regulate signaling cascades that immediately follow activation through the T cell receptor. In this way, oxidant-induced zinc release from MT, in combination with other pathways of zinc mobilization across plasma and vesicular membranes, create dynamic intracellular zinc signals that shape appropriate immune responses. In contrast to transient MT expression, chronic inflammation leads to sustained MT overexpression which is associated with low intracellular zinc ion bioavailability and impaired T cell pathway activation in older populations [66, 67]. These observations underscore a balance where appropriate increases in MT lead to increased zinc signaling, proliferation, and immune function while aberrant overexpression during chronic inflammation leads to decreased zinc signaling and immune depression.

## Conclusions

Previous studies have observed that human CD4^+^ T cells express higher levels of MT than other peripheral blood cell populations and that individual levels of MT induction in lymphocytes varies considerably within the human population [68, 69]. Differential transduction and amplification of zinc signals in CD4^+^ T cells may help to explain the association of MT expression with autoimmune disease, infection and cancer via regulation of CD4^+^ T cell signaling. This is especially true when the consequences of increased MT expression in inflammatory autoimmune disease are divergent. For example, MT is associated with increased inflammation and pathogenesis in inflammatory bowel disease models [70] but suppression of inflammation in models of autoimmune arthritis [35, 71]. The unique contribution of MT to ROS-mediated zinc signal transduction provides further insight into the complex relationship between MT and inflammation and could represent a novel therapeutic target for the manipulation of immune responses.

## Methods

### Mouse strains

All studies were performed using cells from mice bred and housed at the University of Connecticut in accordance with the University of Connecticut Institutional Animal Care and Use Committee (IACUC) guidelines. C57BL/6 J mice (abbreviated MT^+/+^) were backcrossed to a *Mt1Mt2* knockout strain (129S7/SvEvBrd-*Mt1*^*tm1Bri*^*Mt2*^*tm1Bri*^ ) [72] to yield congenic MT knockout mice (C57BL/6-*Mt1*^*tm1Bri*^*Mt2*^*tm1Bri*^ , abbreviated MT^-/-^ ). Mice used in these experiments were between 9 and 24 weeks of age and genotypes were verified by PCR according to protocols provided by Jackson Laboratory (www.jax.org). All mice were age- and sex- matched within each set of experiments.

### Reagents

All reagents were molecular biology grade, reconstituted and stored according to manufacturer’s protocols, and 0.22 μM filter-sterilized before use in cell culture including TPEN (N,N,N′,N′-Tetrakis(2-pyridylmethyl) ethylenediamine), pyrithione (2-Mercaptopyridine *N*-oxide sodium salt), ZnSO_4_ (zinc sulfate), H_2_O_2_ (hydrogen peroxide, 30 % w/v), DTDP (2,2'-dithiodipyridine), PMA (phorbol 12-myristate 13-acetate) (Sigma, St. Louis MO), Fluozin-3 AM, CM-H_2_DCFDA (5(and 6)chloromethyl-2',7'-dichlorodihydrofluorescein diacetate, acetyl ester), and CPM (7-Diethylamino-3-(4'-Maleimidylphenyl)-4-Methylcoumarin) (Life Technologies, Carlsbad, CA).

Labeled antibodies used in flow cytometry included anti-CD4-alexafluor 647 (clone RM4-5), anti-CD4-FITC (clone GK1.5), anti-CD4-PE (clone GK 1.5), anti-CD4-PerCP (clone RM4-5), anti-CD8-PE (clone 53-6.7), anti-CD25a-PE Cy7 (clone PC61), anti-rabbit IgG FITC (BD Biosciences, Franklin Lakes, NJ), and Isotype control IgG-alexafluor647 (mouse IgG1k, clone MOPC21) (Biolegend, San Diego, CA). Anti-MT (mouse IgG1k, clone UC1MT) was produced in-house and labeled with alexafluor647 using an NHS-alexafluor647 labeling kit (Life Technologies, Carlsbad, CA). Unlabeled antibodies included anti-phospho p38, anti-p38 (Cell Signaling, Beverly, MA) and isotype control rabbit IgG (Chemicon, Billerica, MA). Secondary antibody anti-rabbit IgG-HRP was used for western blotting (BD biosciences, Franklin Lakes, NJ). Antibodies used for activation of CD4^+^ T cells included anti-mouse CD3 (clone 17A2) (Biolegend, San Diego, CA) and anti-mouse CD28 (clone 37.51) (BD biosciences, Franklin Lakes, NJ) for plate-bound stimulation, and hamster anti-CD3 (clone 145-2C11) (BD Biosciences, Franklin Lakes, NJ) and anti-hamster IgG (KPL, Gaithersburg, MD) for stimulation in suspension.

### Cell isolation

Spleens were removed aseptically and single cell suspensions were made using frosted glass slides (Corning, Corning, NY) and passage through an 18-gauge needle and a 70 μM cell strainer (Fisher, Morris Plains, NJ). Viable lymphocytes were then enriched by density centrifugation using Histopaque 1083 (Sigma, St. Louis, MO). For CD4^+^ T cell isolation, cells were isolated using a negative selection magnetic isolation column (Miltenyi Biotec, Cambridge, MA) according to manufacturer instructions.

### Media

During splenocyte isolation or during treatment outside of a CO_2_ incubator, mouse cells were maintained in DMEM containing 4.5 g/L glucose, 1 mM sodium pyruvate and 2 mM L glutamine (Gibco, Waltham, MA), supplemented with 10 % FBS (Hyclone, South Logan, UT), 25 mM HEPES buffer, 1 % penicillin/streptomycin (Gibco, Waltham, MA), and 50 μM 2-mercaptoethanol (Fisher, Morris Plains, NJ). For cell culture, including activation and differentiation, cells were cultured in the same media with added 1 % non-essential amino acids (Gibco, Waltham, MA), and without HEPES using 96 or 24 well tissue culture treated plates (Fisher, Morris Plains, NJ) at 10^6^ cells/mL for CD4^+^ T cell culture and 2 x 10^6^ cells/mL for splenocyte culture at 37 °C and 5 % CO_2_. For analysis by flow cytometry, cells were resuspended in Fluorobrite™ DMEM (Gibco, Waltham, MA) with 10 % FBS (Hyclone, South Logan, UT) and 25 mM HEPES.

### Cell activation and differentiation

Mouse splenocytes were incubated in 24 well plates coated with 1 μg/mL anti-CD3 and 1 μg/mL soluble anti-CD28. Tr1 inducing conditions: 50 ng/mL of IL-27 (R&D systems, Minneapolis, MN) was added according to previously described protocols [30]. Th1 inducing conditions: 20 ng/mL IL-2, 20 ng/mL IL-12, and 10 μg/mL anti-IL-4 (R&D systems, Minneapolis, MN) was added. Th0 inducing conditions: no added cytokine. After 5 days, cells were counted and resuspended at 1x10^6^ cells/mL in 75 % fresh cell culture media (without cytokines) to rest for 2 days.

Restimulation of mouse CD4^+^ T cells: Rested cells were plated at 1 x 10^6^ cells/mL in fresh media in 96 or 24 well plates coated with 5 μg/mL anti-CD3 with a brief spin (200 x g for 2 min) to ensure contact between cells and plate bottom. Alternatively, 100 ng/ml PMA was added to wells. At indicated time points, cells were collected, put on ice for 3 min and centrifuged at 300 x g for 5 min at 4 C. Supernatants were stored at -80 C until cytokine analysis by ELISA. For production of ROS, cells were coated with anti-CD3 [10 μg/mL] (BD Biosciences, Franklin Lakes, NJ) on ice and restimulated by addition of anti-hamster IgG [10 μg/mL] in warm media according to previously described protocols [43].

### Measurement of [Zn^2+^]_i_ or ROS

Cells were loaded with Fluozin-3 AM [0.5 μM] in complete media at 37 °C for 30 min. Cells were washed with media and incubated for an additional 20 min at 37 C. Cells were then stained for surface markers at 4 C for 20 min. Cells were washed again and resuspended in Fluorobrite™ media + propidium iodide [2.5 μM] (Life Technologies, Carlsbad, CA) and subdivided into 3 tubes (Fmin, F, Fmax) and stored on ice. 10 min before analysis, an equal volume of TPEN in PBS [100 μM] was added to each Fmin tube, PBS was added to the F tube, and pyrithione [200 μM] + ZnSO_4_ [100 μM] in PBS was added to the Fmax tube.

To measure changes in [Zn^2+^]_i_ or ROS production during stimulation for 0–2 h, cells were loaded with Fluozin-3 AM [0.5 μM] or CM-H_2_DCFDA [1 μM], respectively, and stained with anti-CD4 for the final 10 min of activation. Cells were immediately put on ice, washed 1x with media, and analyzed by flow cytometry.

### Measurement of intracellular thiols

Total intracellular thiols were measured using a N-[4-(7-diethylamino-4-methyl-3-coumarinyl)phenyl]maleimide (CPM) assay (Life Technologies, Carlsbad, CA) as described previously [41]. Briefly, cells were lysed in cell lysis buffer (0.5 % NP-40 + 1 mM EDTA in PBS) and lysate protein concentrations were normalized by BCA assay (ThermoFisher, Waltham, MA). Cell lysates (in triplicate) were immediately incubated with CPM and fluorescence was read on a SpectraMax M2 Plate Reader (ex.387 / em. 465) using softmax Pro software (Molecular Devices, Sunnyvale, CA) and compared with a reduced glutathione standard curve (10 μM–10nM) to quantify intracellular thiols.

### Measurement of p38 MAPK

Tr1 cells were activated by briefly centrifuging cells in media onto 96-well anti-CD3 coated plates [5 μg/ml] or by adding PMA [100 ng/ml] with or without ZnSO_4_ [20 μM], pyrithione [50 μM], or TPEN [50 μM]. Cells were incubated at 37 C for indicated times and then immediately fixed by adding an equal volume of 4 % paraformaldehyde and incubating for 10 min at 37 C. Cells were washed 2x with PBS and stained for CD4. Cells were then permeabilized and stained for phospho-p38 before analyzing by flow cytometry. For western blotting experiments, cells were activated with anti-CD3 [10 μg/ml] and anti-hamster IgG [10 μg/ml] for the indicated time and immediately placed on ice, centrifuged, and lysed in protease/phosphatase inhibitor cocktail (Roche, Indianapolis, IN). Lysate protein concentrations were normalized by BCA assay and separated by SDS-PAGE before being analyzed by western blot for the presence of phospho-p38, total-p38, or tubulin.

### Measurement of secreted cytokines

IL-10 and IFN-gamma were measured using anti-mouse IL-10 and anti-mouse IFN-gamma Duoset antibody kits (R&D Systems, Minneapolis, MN) and Immulon H2B 96 well plates (Fisher, Morris Plains, NJ). Plates were read on a SpectraMax Plate Reader using softmax Pro software (Molecular Devices, Sunnyvale, CA).

### Data analysis

Data was analyzed using Graphpad Prism (v6). Comparisons between 2 groups were made using independent two-tailed T tests and a Sidak-Bonferroni correction for multiple comparisons where appropriate or 1-way ANOVA for comparisons of 3 groups. Where Linear Regression analysis was used, the slopes of best-fit lines were compared to determine p values.

## Abbreviations

[Zn^2+^]_i_, intracellular labile zinc concentration; CM-H_2_DCF, chloromethyl dichlorodihydrofluorescein; DTDP, dithiodipyridine; FasL, Fas Ligand; FBS, fetal bovine serum; GSH, reduced glutathione; GSSG, glutathione disulfide; H_2_O_2_, hydrogen peroxide; IgG, immunoglobulin G; MAPK, mitogen-activated protein kinase; MT, metallothioneins; PMA, phorbol 12-myristate 13-acetate; ROS, reactive oxygen species; STAT, signal transducer and activator of transcription; TcR, T cell receptor; Th0, T-helper cell type 0; Th1, T-helper cell type 1; TPEN, N,N,N',N'-tetrakis(2-pyridylmethyl)ethane-1,2-diamine; Tr1, type-1 regulatory T cell; Zip, Zrt- and Irt-like proteins; ZnT, zinc transporters.

## Additional files

Additional file 1: Figure S1. Increasing extracellular [Zn2+] does not affect intracellular Zn2+ release following exposure to ROS. Tr1 cells from metallothionein wildtype control mice were exposed to 5μM H2O2 in media (circle) or media with 20μM ZnSO_4_ added (square). Baseline Fluozin-3 fluorescence was established for 30 s followed by a 7 min exposure to H_2_O_2_. (PDF 109 kb) Additional file 2: Figure S2. T helper cell inducing conditions have an effect on the intracellular [Zn^2+^] of CD4^+^ T cells during primary activation and differentiation. Splenocytes from MT knockout (MT^-/-^) (*n* = 3–4) or wildtype controls (MT^+/+^) (*n* = 3–4) were stimulated with anti-CD3 and anti-CD28 for 6 days in the presence of no added cytokines (Th0), IL-12 + IL-2 + anti-IL-4 (Th1), or IL-27 (Tr1) to promote differentiation and expansion of T helper cell populations. Intracellular [Zn^2+^] in the CD4^+^ T cell population was measured for each inducing condition and MT genotype at +6 days post stimulation. (PDF 90 kb) Additional file 3: Figure S3. MT gene dose does not affect total p38 MAPK levels in CD4^+^ Tr1 cells. CD4^+^ Tr1 cells from MT knockout (MT^-/-^) or wildtype control (MT^+/+^) mice were stimulated with anti-CD3 cross-linked with anti-IgG for 15–120 min or with no cross-linking (timepoint 0). Cell lysates were analyzed for the presence of total p38 or tubulin by SDS-PAGE and western blotting. (PDF 68 kb) Additional file 4: Figure S4. Addition of ZnSO_4_ [20μM] to cell culture media for 24 h increases intracellular [Zn^2+^] in CD4^+^ Tr1 cells. Tr1 cells from MT knockout (MT^-/-^) (*n* = 6) or wildtype control (MT^+/+^) mice (*n* = 6) were incubated in media + ZnSO_4_ [20μM] or media alone for 24 h. Tr1 cells were loaded with fluozin-3 AM and the intracellular [Zn2+] was measured by flow cytometry. (PDF 98 kb)
