# Supplementary figures and images for: Metallothionein regulates intracellular zinc signaling during CD4+ T cell activation
Source: BMC Immunol. 2016 Jun 2;17:13. doi: 10.1186/s12865-016-0151-2 (PMC4890327; doi:10.1186/s12865-016-0151-2)

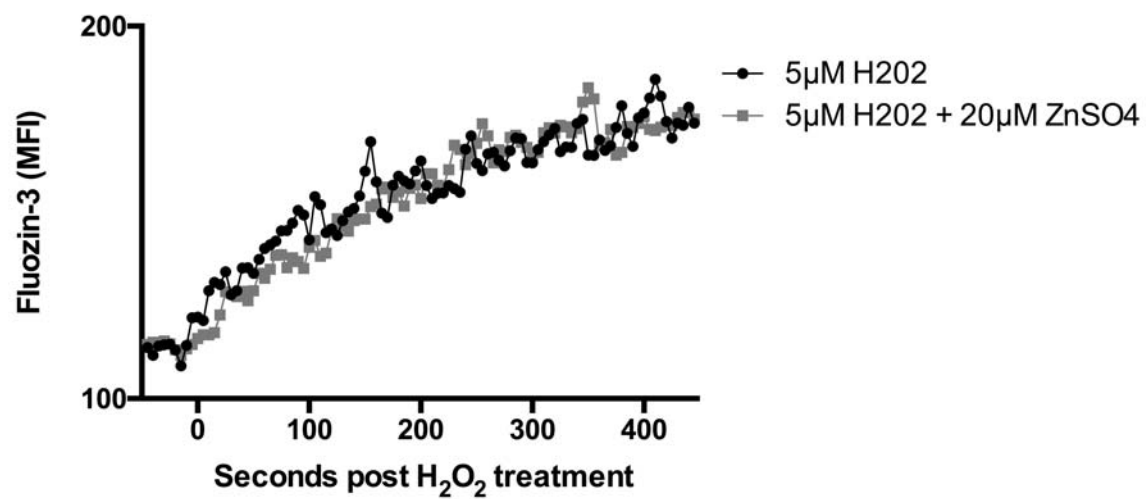

Supplement: Additional file 1: Figure S1. — Increasing extracellular [Zn2+] does not affect intracellular Zn2+ release following exposure to ROS. Tr1 cells from metallothionein wildtype control mice were exposed to 5μM H2O2 in media (circle) or media with 20μM ZnSO4 added (square). Baseline Fluozin-3 fluorescence was established for 30 s followed by a 7 min exposure to H2O2. (PDF 109 kb) [file 12865_2016_151_MOESM1_ESM.pdf]

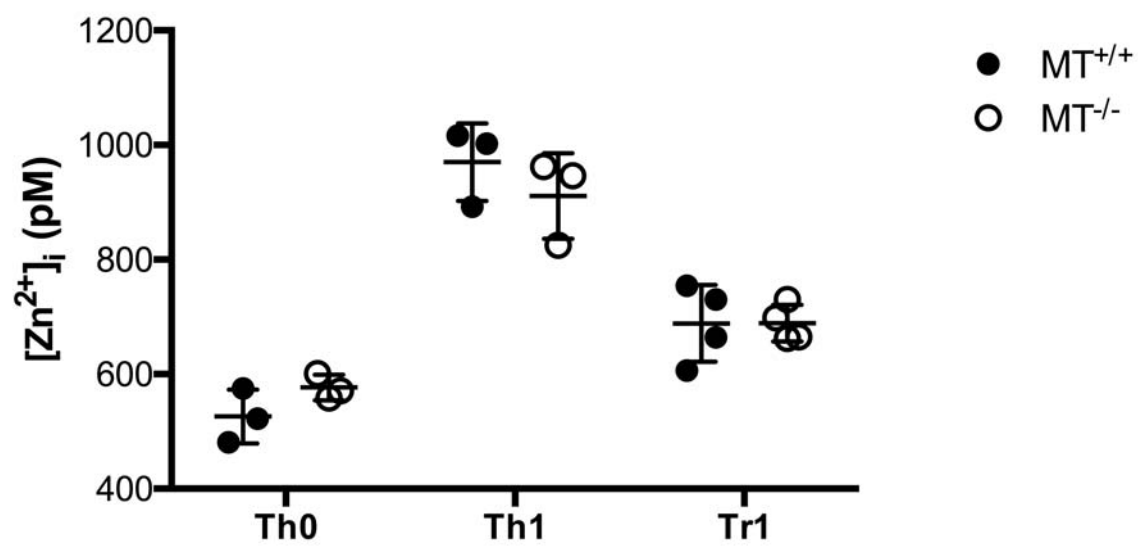

Supplement: Additional file 2: Figure S2. — T helper cell inducing conditions have an effect on the intracellular [Zn2+] of CD4+ T cells during primary activation and differentiation. Splenocytes from MT knockout (MT-/-) (n = 3–4) or wildtype controls (MT+/+) (n = 3–4) were stimulated with anti-CD3 and anti-CD28 for 6 days in the presence of no added cytokines (Th0), IL-12 + IL-2 + anti-IL-4 (Th1), or IL-27 (Tr1) to promote differentiation and expansion of T helper cell populations. Intracellular [Zn2+] in the CD4+ T cell population was measured for each inducing condition and MT genotype at +6 days post stimulation. (PDF 90 kb) [file 12865_2016_151_MOESM2_ESM.pdf]

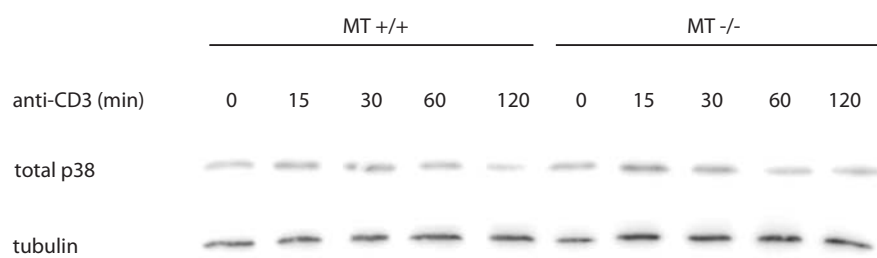

Supplement: Additional file 3: Figure S3. — MT gene dose does not affect total p38 MAPK levels in CD4+ Tr1 cells. CD4+ Tr1 cells from MT knockout (MT-/-) or wildtype control (MT+/+) mice were stimulated with anti-CD3 cross-linked with anti-IgG for 15–120 min or with no cross-linking (timepoint 0). Cell lysates were analyzed for the presence of total p38 or tubulin by SDS-PAGE and western blotting. (PDF 68 kb) [file 12865_2016_151_MOESM3_ESM.pdf]

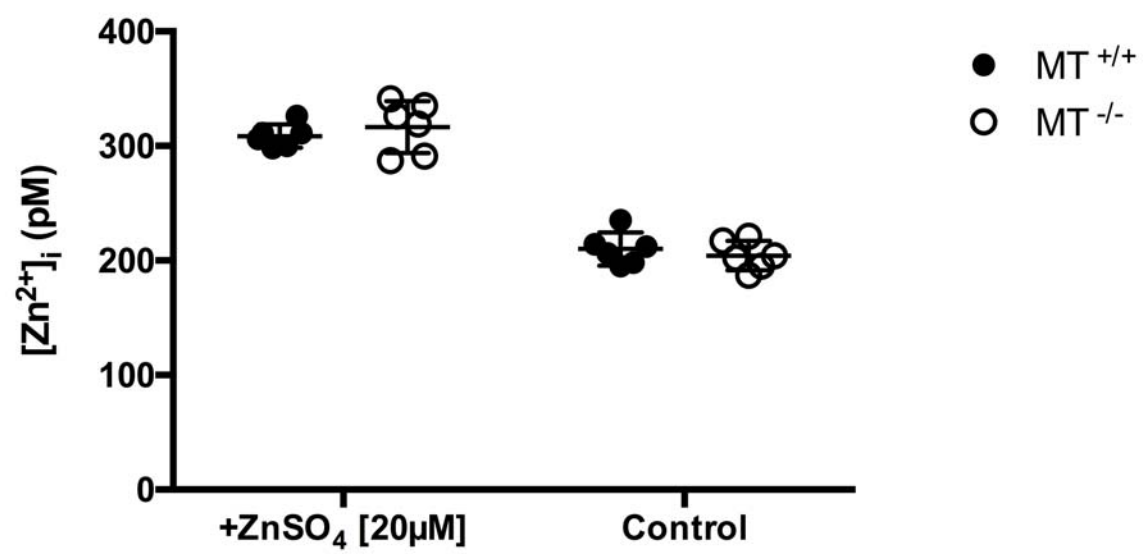

Supplement: Additional file 4: Figure S4. — Addition of ZnSO4 [20μM] to cell culture media for 24 h increases intracellular [Zn2+] in CD4+ Tr1 cells. Tr1 cells from MT knockout (MT-/-) (n = 6) or wildtype control (MT+/+) mice (n = 6) were incubated in media + ZnSO4 [20μM] or media alone for 24 h. Tr1 cells were loaded with fluozin-3 AM and the intracellular [Zn2+] was measured by flow cytometry. (PDF 98 kb) [file 12865_2016_151_MOESM4_ESM.pdf]
